# Posttreatment health interventions for adult cancer survivors and their families: an integrated review

**DOI:** 10.1007/s00520-024-08909-1

**Published:** 2024-10-08

**Authors:** Elisabeth Coyne, Karin B. Dieperink, Barbara Voltelen, Mayckel da Silva Barreto, Cristina Garcia-Vivar

**Affiliations:** 1 Griffith University, Brisbane, Australia; 2 Family Focused Healthcare Research Center (FaCe), Department of Clinical Research, University of Southern Denmark, J.B. Winsløws Vej 19.3, 5000 Odense C, Denmark; 3 Research Unit of Oncology, Odense University Hospital, Sdr. Boulevard 29, 5000 Odense C, Denmark; 4 Department of Nursing Education and Health Sciences Research Center, University College Lillebaelt, Vejle, Denmark; 5 Nursing Department, State University of Maringá, Postgraduate Nursing Program, Maringá, Paraná, Brazil; 6 Department of Health Sciences, Public University of Navarra, Pamplona, Spain; 7 Institute for Health Research of Navarra (IdiSNA), Pamplona, Spain

**Keywords:** Long-term cancer survivors, Adult cancer, Family, Survivorship care plans, Interventions

## Abstract

**Purpose:**

This review aimed to synthesize the literature regarding health interventions delivered to adult cancer survivors and their families during posttreatment phase.

**Methods:**

An integrative literature review was conducted that included quantitative and qualitative studies. The search was carried out in four databases using the same terms or MeSH terms and included data from January 2012 to February 2024. After quality assessment, data were extracted and synthesized. The protocol was registered in PROSPERO.

**Results:**

Among the seven studies included, two studies were randomized controlled trials, three were observational, and two utilized a qualitative approach. The studies originated from France, Australia, Canada, the UK, and the USA. In total, 704 participants were included, 294 were cancer survivors, 40 were non-cancer patients, 271 were family and caregivers, and 99 were healthcare professionals. The studies assessed survival durations after cancer treatment, ranging from 18 months to 6 years. The sparse interventions found across the studies used a multifaceted approach tailored to address various aspects of cancer survivorship and caregiver support.

**Conclusion:**

This review provides insights into the complex landscape of posttreatment support requirements for cancer survivors and their family caregivers. This finding underscores the critical necessity for additional intervention research involving comprehensive, accessible, and supportive services that address the multifaceted dimensions of survivorship for the patient and family as a unit.

## Introduction

Improvements in the diagnosis and treatment of cancer have led to a greater number of adult cancer survivors who live with a changed level of health [1]. Cancer survivors have been found to suffer from posttraumatic distress [2] and continued late effects of the cancer and treatment [3]. Research has shown that lifestyle choices and comorbidities can adversely affect long-term survival and quality of life among adult cancer survivors [1, 4], emphasizing the need for the promotion of self-management and survivorship plans to reduce the risk of poor quality of life and the need for continued healthcare [4, 5]. Additionally, the impact of cancer survivorship extends beyond the individual, affecting the dynamics and well-being of their families [6], and emphasizing the importance of comprehensive support systems and resources for survivors and their loved ones.

### Background

The term survivorship denotes a period when you should be celebrating your escape from death and a time when survivors experience a sense of positive and negative concepts [7, 8]. This is a period of adjustment to a new level of normal, dealing with the late effects of treatment [1, 9]. The long-term late effects of cancer treatment can range from changes in hair growth, to more complex health, and psychological changes [3]. During cancer treatment, patients have unmet physical, psychological, social, and spiritual needs [10], and into the survivorship stage, these unmet needs influence patient and family connections and communication within the healthcare system [2, 11]. Cancer survivors may suffer from chronic pain, persistent fatigue, cognitive impairments, organ-related side effects, emotional struggles such as depression and fear of cancer recurrence, along with financial and insurance-related burdens, and other conditions [12].

The diagnosis and treatment of cancer will always be a family affair [10, 13], but the family is particularly important in the survival phase when there may be little connection with the healthcare system [14, 15]. From a broad and holistic perspective, “family,” is who its members say they are and together they form a system that adapts to its functioning [16]. The family will support the cancer survivor to find a new level of normal, working with the health system to find the right level of health services and continuation of care [4]. The family continues to provide support to the person with cancer, managing long-term side effects [17]. Providing ongoing support influences the family members own health outcomes, leading to high levels of distress, unmet needs, and chronic disease, highlighting the need for family-centered health interventions in the posttreatment period [13, 18].

Although there is evidence that cancer survivors need long-term follow-up to assist their adjustment posttreatment, there is still little integration of survivorship care plans or survivorship clinics [4, 19]. The needs of cancer survivors are often lost during the transition between active treatment and follow-up care [20]. Currently, many cancer survivors describe oncological follow-up as primarily focused on the efficacy of their cancer treatment and detection of relapse, and less on addressing potential long-term effects caused by their treatment [19, 21, 22].

While debates over the definition of the term “cancer survivor” have long been ongoing, there continues to be controversy over its application [12]. Researchers and government organisations use the term, cancer survivor, to refer to individuals at any stage of their cancer journey, from diagnosis onward [23]. Although, it is most commonly recognized as someone who has successfully completed treatment, achieved a state of disease-free health, and may subsequently grapple with a spectrum of challenges inherent to cancer survivorship [17]. The term cancer survivor also extends to include family members, caregivers, and loved ones who are affected by the cancer diagnosis and its aftermath, recognizing the broader impact of the disease on their lives [17]. In this distinct phase following the end of active treatment, a “short-term cancer survivor,” is a person who has completed treatment within the last 1 to 5 years and is currently in remission with no signs or symptoms of cancer. Conversely, a “long-term cancer survivor,” is someone who successfully completed treatment at least 5 years ago [17, 24]. Regardless of the circumstances, it remains imperative to gain a deep understanding of the ongoing journeys of cancer survivors and their families. This comprehension is pivotal for delivering all-encompassing care finely tuned to address the patient and families unique and specific needs. There is a lack of knowledge about posttreatment experiences and needs for adult cancer survivors, including their families. To develop new ideas to support future cancer survivorship, a review of the current practices and interventions for adult patients and families in the survivorship phase needs to be conducted. Therefore, this review aimed to synthesize the literature regarding health interventions delivered to adult cancer survivors and their families during the posttreatment phase.

Before continuing, it is imperative to contextualize the concept of posttreatment intervention within this review to ensure a nuanced understanding of the diverse contextual factors influencing the applicability of such interventions for cancer survivors and their families. Building on the findings of other authors in cancer survivorship, such as Pimentel-Parra, Soto-Ruiz [25], the focus of this review was research that explored survivorship at least one year after the end of primary or active treatment. Furthermore, the term “interventions” was intended to encompass a broad range of posttreatment strategies and supports for cancer survivors and their families, involving the assessments, and interventions that address the psychological, social, personal, or relational adjustment needs related to cancer. This definition encompasses not only the provision of assistance, support, or treatment to meet the physical and psychosocial needs of cancer survivors and their families but also involves the understanding of the users’ perspectives and preferences on survival care.

The central research question guiding this study was: What is the current knowledge of research on posttreatment health interventions for adult survivors of cancer and their families?

Specifically, we aimed:

1. To synthesize the research on adult cancer survivorship in the posttreatment phase (starting from one year after the end of active treatments)
2. To identify current interventions, length of time, and who was involved
3. To identify research gaps

## Method

An integrative literature review was conducted, enabling quantitative and qualitative studies to be included [26]. The steps in an integrative review are to define the topic and develop the research question, inclusion and exclusion criteria and the development of Population, phenomena of interest, and context (PPC) to structure the search [27]. This review was registered in Prospero [CRD42021229080].

### Search strategy

The search was conducted in March 2024 across several databases using the same terms or MeSH terms with data inclusion from January 2012 to February 2024 to include current practices and trends in survivorship. The following databases were used: Medline (PubMed), CINAHL (EBSCO Nursing and Allied Health), the Cochrane Library, and LILACS (Latin American and Caribbean Health Sciences Literature), with all the records downloaded into Covidence (www.covidence.org), to enable a systematic review across all the team members. Furthermore, we consulted the Index to Theses, CAPES Bank of Dissertations and Theses, Networked Digital Library of Theses and Dissertations, and New York Academy of Medicine Gray Literature Report to find relevant theses, but none were found. A health librarian was used to ensure that the MeSH and key search terms obtained met our inclusion criteria. See Table 1 displays the search strategy used within the abstract and their combinations.

**Table 1:** Search terms used in the abstract and their combinations

AB (Cancer survivors OR Long-Term Cancer Survivors* OR Adult cancer OR Family OR Caregiver OR Family members Partner OR Spouses OR Husband OR Daughters OR Sons OR Relative OR Adult child* OR Next of kin OR wives) AND AB ( Survivorship Care plans OR Survivorship OR Supportive care OR Nurse-led interventions OR Self-management OR Rehabilitation OR Follow-up) AND AB (Community care OR General practice OR Survival clinics OR Primary health care OR Cancer care OR Follow-up care) AND AB (Cancer post treatment OR Management of late effects OR Late adverse effects OR Side effects Survivorship OR Fear of recurrence OR Quality of life)	

### Selection criteria

The first screening for inclusion and exclusion criteria within the title and abstract was completed by double screening within Covidence by the authors BV and EC. Once downloaded into Covidence, duplicates were removed. All the authors independently reviewed the full texts of the articles against eligibility criteria with a double-screening rule within Covidence. The final references and PDFs were downloaded into Endnote (version 20). See Table 2 presents the inclusion and exclusion criteria of this review.

**Table 2:** Inclusion and exclusion criteria used to identify primary studies

Inclusion criteria	Exclusion criteria
Adult cancer survivors (≥ 18 years of age with any cancer diagnosis) at least 1 year after the completion of active cancer treatment (i.e., surgery, chemotherapy, radiotherapy, immunotherapy)	Not related to cancer survivorship (under a 1-year posttreatment)
The sample should consist of both survivors and their family (e.g., partner, caregiver, adult children, parent, or relative)	Not including the family
Focusing on a healthcare intervention or promoting practice or support of both cancer survivors and their family. Also involves having an understanding of the users’ perspective and preferences on survival care	Studies presenting joint results of cancer patients undergoing treatment and cancer survivors in the posttreatment phase, but the data are not divided into separate categories
Primary research (qualitative, quantitative or mixed methods) and full-text available	Exploring palliative care
Written in English, Portuguese, Spanish, French, or Danish, considering that the authors were fluent in these languages	If they enrolled paediatric survivors

### Data extraction and synthesis

The data extraction was completed by author EC into an Excel document. The data were extracted across a range of variables, including author, year, country, title, aim, design, method, participants, survival length of time since treatment completion, measurements used, interventions/care, recommendations, and main findings. The research team reviewed and addressed any ambiguity, reconciled differing interpretations, and adjusted the data extraction spreadsheet. A content analysis was conducted to identify themes within the included articles to provide an overview of the current knowledge regarding interventions and support in cancer survivorship care [28]. The findings were summarized and presented narratively.

### Quality assessment

The reviewers assessed methodological rigor of the included studies using the Mixed Methods Appraisal Tool (MMAT) – Version 2018, which allows a framework for critical appraisal of mixed methodological studies [29]. The quality appraisal score for each article is presented numerically with descriptors, and a score of 75% or greater (quality appraisal score range: 0–100%) was considered high quality. The relative quality of each article is reported, with none being excluded based on a poor-quality assessment [29].

## Results

### Search results

**Fig. 1:**
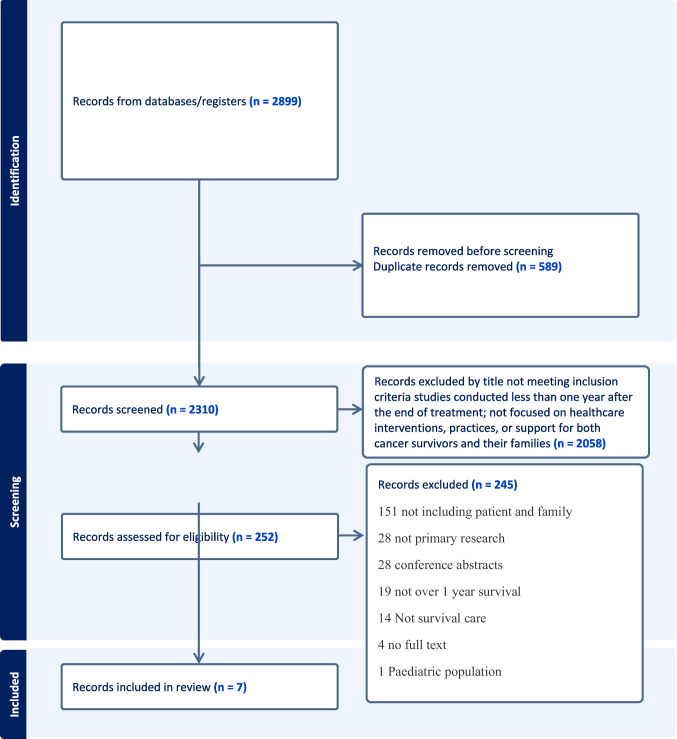
Prisma flow diagram

The initial database search yielded 2899 articles, and 589 duplicates were removed after import via Covidence. The first screening via title and abstract removed 2058 articles that did not meet the inclusion criteria. After screening the full texts, 252 additional articles were excluded. The final seven articles met the inclusion criteria. See Fig. 1 “Prisma flow diagram” of identified, screened, included, and excluded studies. See Table 3 “Summary of included studies.”

**Table 3:** Summary of included studies

Author, year, country	Title	Aim	Design Methodology	Participants Time since treatment (mean duration)	Measurement Outcome measures	Intervention/practice	Main findings	Appraisal score
Cantisano et al., 2021, France	Patient-reported functional executive challenges and caregiver confirmation in adult brain tumor survivors	To provide further information concerning the validity of patient-reported executive function (EF) in survivors of primary brain tumor (PBT) compared with a report provided by each patient’s caregiver	Quantitative observational study The methodology involved assessing functional executive disorders in survivors of primary brain tumors and non-cancer controls by comparing self and informant EF reports	. 40 brain cancer survivors between 20 and 59 years 40 non cancer patients 37 family caregivers of brain cancer survivors 31 family caregivers of non-cancer participants Over 36 months	Mini-Mental State Examination (MMSE) Behavior Rating Inventory of Executive Function for Adults (BRIEF_A) Behavioral Regulation Index (BRI) Metacognition Index (MCI) Validity of patient-reported executive function (EF)	Self-report of patients about their executive behavior problems relating to the performance of daily-life activities and caregivers-rated reports about the survivors´ executive behavior	. Cancer survivors showed higher difficulties compared to the non-cancer participant on executive difficulties associated with the cognitive late effects of brain cancer. Survivors suffer from a negative impact of these functional executive challenges when carrying out activities of daily living. The difficulties are in emotional control, working memory, and self-monitoring. Correspondingly, a high level of concordance was observed between self- and caregiver- ratings of executive functions difficulties. Family caregivers´ perceptions coincided with the survivors’ perceptions of challenges in daily life	83%
Gunn et al., 2021, Australia	Improving survivors’ quality of life post-treatment: the perspectives of rural Australian cancer survivors and their carers	To provide deeper understanding of the experiences of rural survivors who have completed active cancer treatment and returned to their rural communities, and (b) determine strategies to re-orient existing services or develop new interventions to meet rural survivors’ service preferences and needs	Qualitative study Essentialist/realist approach with inductive analysis of qualitative data, thematic analysis	13 survivors completed active treatment six caregivers three were both patients and caregiver experience 18 months	Not applicable Experiences of cancer survivors in rural communities’ posttreatment and challenges in engaging with quality of life-related support services	Telephone or face-to-face services for programs designed to improve quality of life, proactive support reaching out to participants, continuity of care with the same person, nurses delivering posttreatment support, and telehealth for reduced travel burden	A range of physical, psychological, and practical challenges. Participants highlighted that the completion of treatment does not signal the end of the negative impact of cancer on their lives (long-term physical side effects after cancer treatment that affect their quality of life; new psychological challenges; frustration due to unmet expectations about their return to pre-cancer quality of life; reduced levels of independence; isolation; lacking a clear posttreatment care pathway and information). There is a lack of trust in local rural healthcare services; a lack of clear posttreatment pathways to quality of life-enhancing support services; long waiting times to see GP; difficulty understanding their GP; lack of availability of relevant local services and a lack of information and direction on what is available and how services may be able to help. Most posttreatment quality-of-life-focused support is provided by family, Friends, nurses (visiting and via structured telephone-based support programs) and support groups acceptable strategies to overcome barriers included nurse-led, telephone-based, or face-to-face interventions, initiated and continued by the same service provider, and that included support to manage emotional challenges associated with posttreatment survivorship	100%
Lambert et al., 2022, Canada	Feasibility, acceptability, and clinical significance of a dyadic, web-based, psychosocial and physical activity self-management program (TEMPO) tailored to the needs of men with prostate cancer and their caregivers: a multi-center randomized pilot trial	To conduct a pilot study of TEMPO—the first web-based, personalized dyadic program designed to support psychosocial well-being and physical activity management for men with prostate cancer and their caregivers	Multi-center, stratified, 1:1 parallel, two-group, pilot RCT Involved recruiting 49 men with prostate cancer and their caregivers, randomizing them to TEMPO or usual care, and using baseline and follow-up questionnaires to assess feasibility, acceptability, and clinical significance. Recruitment was done through various methods, and data collection included online questionnaires in French and English	49 survivors of prostate cancer 49 caregivers 24 months	Hospital Anxiety and Depression Scale (HADS); Short-Form Health Survey (SF-12); Perceived Stress Scale (PSS); Health Education Impact Questionnaire (heiQ v3.0); Health Literacy Questionnaire (HLQ); International Physical Activity Questionnaire-Short Form (IPAQ-SF); Physical Activity Plan and Intention; Multidimensional Self-Efficacy for Exercise Scale (MSES); Dyadic Coping Inventory (DCI); Revised Dyadic Adjustment Scale (RDAS); healthcare services and change in employment (nine items) questionnaire; System Usability Scale (only T2) Feasibility, acceptability, anxiety, quality of life	TEMPO is an evidence-informed program designed for men with prostate cancer and their caregivers (as a dyad) to learn self-management skills based on their priority needs, set goals together, and build confidence to address and manage their needs. Alongside the psychosocial self-management content (e.g., managing stress, symptoms), TEMPO supports the use of physical activity as a self-management strategy to enhance overall physical (and mental) health. Briefly, TEMPO is a 10-week, web-based intervention, where men with prostate cancer and their caregivers are guided through five modules: needs assessment, goal setting and action planning based on most pressing needs, coping planning, sources of support and motivational tools, and celebrating successes achieved through TEMPO	Participants reported clinically significant improvements in anxiety (effect size = 0.24). Anxiety decreased by the minimal clinically important difference in 42.9% of patients and 44.4% of caregivers using TEMPO, exceeding rates in the control group (39.1% patients and 30.8% caregivers, odds ratio = 1.48) Increases in mental quality of life were observed in 33.3% of patients; for quality of life-physical, twice as many caregivers in TEMPO improved (33.3% caregivers) than in the control group (16.5%) A trend is noted in the separate effect sizes for patients and caregivers, whereby caregivers seemed to benefit more from TEMPO than patients on mental health outcomes	83%
Marshall-McKenna et al., 2022, UK	A multinational investigation of healthcare needs, preferences, and expectations in supportive cancer care: co-creating the LifeChamps digital platform	To evaluate healthcare needs, preferences, and expectations in supportive cancer care as perceived by cancer survivors, family caregivers, and healthcare professionals	Descriptive, cross-sectional, multi-methods study Cross-sectional descriptive multi-methods study with recruitment of participants through online surveys or telephone interviews	70 cancer patients 23 caregivers 62 health professionals 25 months	Charlson comorbidity index Perception and acceptance of a digital platform for cancer survivors, caregivers, and healthcare professionals	Healthcare professionals preferred 'on-demand' information updates before patient visits or every 3–4 months. They supported the use of patient-reported outcome measures like EQ-5D-5L, FACT, and the distress thermometer for better treatment. They emphasized the need for clear pathways on acting upon information and user-friendly technology	Cancer survivors and family caregivers’ needs included information and support on practical/daily living, as frustration was apparent with the lack of follow-up services Cancer survivors most frequently identified “finding a new normal” as their priority in life as the consequences of cancer had a varying degree of impact on patients’ lives. Most family caregivers felt that they had returned to normal and were enjoying and appreciating family life, but for some, their caring responsibilities remained despite their family member finishing cancer treatment, and they were still feeling emotional Cancer survivors and family caregivers generally felt that the digital platform would be useful for timely personalized support and aided communication	86%
Payne et al., 2019, USA	Patient and caregiver perceptions of lymphoma care and research opportunities: a qualitative study	To determine care needs and research priorities	Qualitative descriptive Focus groups and individual telephone interviews with survivors and caregivers. Semi structured interviews with a codebook for coding and theme development	eight lymphoma survivors (phase 1) seven caregivers (phase 01) 19 patients (phase 2) 19 caregivers (phase 2) 60 months	Not applicable Quality of life, communication, and emotional well-being,	The study participants received interventions related to discussing their lymphoma experiences, engaging in coping strategies, strengthening social support networks, and utilizing support systems for comfort and care	The majority of participants felt disconnected from their clinical care team due to a lack of communication Focus group participants noted a lack of information regarding diagnoses, treatment, research, and survivorship care. Participants coped with fear through strong social support and fostering relationships with their clinical care teams. Some caregivers felt completely ignored by clinicians. Participants expressed interest in research but had difficulty finding relevant studies. Several interviewees desired holistic, quality of life and survivorship-oriented research and more studies regarding quality of life and mental health Many participants were especially willing to participate if the studies were not invasive or time-consuming	100%
Perz et al., 2013, Australia	Constructions of sex and intimacy after cancer: Q methodology study of people with cancer, their partners, and health professionals	To explore the complex perspectives that people with personal and professional experience with cancer hold about sexuality in the context of cancer	Interview study using Q methodology A set of 56 items was developed based on themes emergent in the data sources, ensuring coverage and balance. The final Q set was reviewed for content and face-validity by oncology clinicians and consumer representatives. This methodology allowed for the exploration of complex perspectives on sexuality post-cancer	44 adult patients with different cancer aged from 20 to 77 years 35 partners 37 health professionals (nine doctors, 11 nurses, 10 psychologists and seven social workers) 69% of patients in remission with treatment completed 28% in active treatment 3% bereaved partners	Not applicable Quality of life and relationship satisfaction achieved through communication and non-genital intimacy post-cancer	Explore the complexity of perspectives on sexuality and intimacy post-cancer, emphasizing the importance of open communication, support, and normalization of a wide range of sexual practices, with practical implications for cancer care and survivorship	Factor 1: Emphasizes communication about sex and intimacy in the context of cancer, with health professionals discussing the effects of cancer on sexual relationships. It rejects the idea that sexual relationships are too personal for health professionals to discuss Factor 2: Focuses on normalizing the experience of sex after cancer through the renegotiation of sex and intimacy. It highlights the importance of open communication within couples for a satisfying sexual relationship . Factor 3: Stresses the importance of intimacy in relationships post-cancer, even if sex may not be wanted or possible. It emphasizes that quality of life and relationship satisfaction can be achieved through communication and non-genital intimacy	100%
Winters-Stone et al., 2016, US	Benefits of partnered strength training for prostate cancer survivors and spouses: results from a randomized controlled trial of the exercising together project	To report the feasibility of a novel couple-based approach to exercise training for couples coping with PC	A 6-month single-blind RCT comparing partnered strength training /couple) to usual care, with assessments including body composition, muscle strength, physical function, and self-reported measures. Inclusion criteria for participants were specified, and feasibility and preliminary efficacy were evaluated through various outcome measures	64 prostate cancer survivors 64 partners 72 months	Body composition (lean, fat, and trunk fat mass in kg, and % body fat), upper and lower body muscle strength by 1-repetition maximum, physical function by the physical performance battery (PPB), self-reported physical and mental health summary scales, physical function, and fatigue subscales of the SF-36, and physical activity with the CHAMPS questionnaire	Partnered strength training intervention called “exercising together” involving 1-h group exercise sessions twice weekly for 6 months, aimed at improving upper and lower body strength, muscle mass, and physical performance for both prostate cancer survivors and their spouse caregivers	. Men became stronger in the upper body (p < 0.01) and more physically active (*p* < 0.01) than usual care. Women increased muscle mass (*p* = 0.05) and improved upper body strength (p < 0.01) and physical performance battery scores (*p* = 0.01) more than usual care	100%

### Characteristics of the included studies

The seven studies included in the review originated from diverse locations, with one conducted in France, two in Australia, one in Canada, one in the UK, and two in the USA (Table 3). Five studies used a quantitative design, two randomized controlled trials and three observational studies. Additionally, two studies utilized a qualitative approach.

In total, 704 participants were included, 294 were cancer survivors, 40 were noncancer patients, 271 were family and caregivers, and 99 were healthcare professionals. The participants in this review encompassed a diverse range of cancer diagnoses, including brain cancer, prostate cancer, and lymphoma, alongside individuals with other types of cancer (not specified, simply referred to as patients with different cancers). The studies examined a range of survival durations following completion of cancer treatment, spanning from 18 months to 6 years.

In terms of subject matter, the included articles cover a wide range of topics, including cognitive changes after brain cancer, rural communities and support services, quality of life in cancer survivorship, acceptance of digital platforms in posttreatment care, intimacy post-cancer, and benefits of exercise after cancer treatment (Table 3).

### Interventions for *cancer* survivors and families

This review found two studies [30, 31] that presented two interventions for survivors and their families. The TEMPO program, which is specifically designed for men with prostate cancer and their caregivers, offers a comprehensive self-management curriculum encompassing psychosocial coping strategies and physical activity regimens [30]. Interventions targeting anxiety and quality of life demonstrated clinically significant improvements for both patients and caregivers, with promising adherence rates and positive outcomes across secondary measures [30].

Partnered strength training initiatives like “Exercising Together” was a valuable intervention for survivors and caregivers that emphasize holistic approaches to address the physical, psychological, and interpersonal challenges associated with cancer survivorship [31]. This intervention yielded tangible physical improvements, including increased muscle strength and activity levels, underscoring the holistic benefits of exercise in cancer survivorship. The intervention reflected a nuanced understanding of survivor and caregiver needs, striving to enhance overall well-being and quality of life throughout the cancer journey.

### Initiatives for posttreatment experiences of *cancer* survivors and their families

Five initiatives aimed at enhancing the posttreatment experiences of cancer survivors and their families were found. These initiatives encompass a range of strategies, including cognitive assessment and support, delivery of supportive services through telephone or face-to-face interactions, advocacy for improved communication and integration of patient-reported outcomes in healthcare decision-making, and exploration of lymphoma survivorship experiences.

The study by Cantisano et al. [32] focused on evaluating cognitive changes through self-reports from patients and caregiver-rated reports, shedding light on survivors’ cognitive functioning and its impact on daily activities. Compared to noncancer participants, brain cancer survivors had greater difficulties in cognitive functions, including cognitive flexibility, attentional control, and working memory. These cognitive late effects of brain cancer significantly impact the everyday activities and overall cognitive functioning of survivors, a fact reported by survivors themselves and corroborated by their caregivers [32].

Telephone or face-to-face services were utilized to deliver programs designed to improve the quality of life of survivors and their families, these programs involved proactive support, continuity of care with healthcare professionals, and the integration of telehealth to mitigate travel constraints [33]. Survivors and their families expressed a distinct preference for engaging with telephone or face-to-face services, especially from nurses, as they addressed their needs and concerns, contributing to enhancing their quality of life [33].

Healthcare professionals advocated for “on-demand” information updates and the integration of patient-reported outcome measures to inform treatment decisions effectively [34]. Healthcare professionals advocated for streamlined information updates and the utilization of patient-reported outcome measures to enhance treatment efficacy [34].

Other interventions delved into the complexities of lymphoma survivorship experiences [35]. Participants conveyed feelings of disconnection from their clinical care teams, primarily due to inadequate communication. Many also expressed a dearth of information regarding their diagnoses, treatment options, and survivorship care after the end of treatment. Coping mechanisms included relying on robust social support networks and fostering relationships with clinical care teams. Interestingly, some caregivers reported feeling neglected by clinicians. While participants expressed an interest in participating in research, they encountered challenges in accessing relevant studies. Overall, there was a desire for more holistic and survivorship-oriented research, particularly focusing on quality of life and mental health aspects posttreatment. The study findings underscore unmet needs in both clinical care and patient-oriented research, highlighting the importance of addressing issues such as posttreatment quality of life, communication gaps between patients and the scientific community, and emotional well-being. These insights can inform healthcare professionals in delivering tailored care, supportive services, and research initiatives that effectively cater to the needs of lymphoma survivors and their caregivers.

Finally, the outcomes of the study by Perz et al. [36] underscore the significance of initiatives addressing sexuality and intimacy post-cancer, emphasizing the critical role of open communication, normalization of sexual experiences, and recognition of non-genital intimacy as pivotal factors in fostering relationship satisfaction and overall well-being among cancer survivors and their partners [36].

## Discussion

This review identified only seven primary studies, revealing a notable gap in evidence concerning effective support for cancer survivors and their families as a unit of care during the posttreatment phase. A systematic review that investigated couple-based interventions revealed improved communication, psychological distress, and relationship functioning during the treatment phase of cancer [37, 38]. Therefore, the authors concluded that despite the underutilization of couple-based interventions, prioritizing further efforts to facilitate their integration into routine practice is essential to reduce distress and enhance coping and adjustment to cancer diagnosis or symptoms. Based on these results, it is imperative to prioritize posttreatment care and information that comprehensively addresses the needs of both survivors and their families as a unit of care, also in the long-term survivorship. This review has also revealed the lack of studies focusing on initiatives or interventions tailored for long-term cancer survivors and their families, underscoring a critical gap in the literature. Recognizing that cancer survivorship is a family affair [17], there is a critical need to enhance the quality of life for survivors and their families by providing comprehensive support and guidance tailored to their unique needs. However, current evidence is insufficient for providing guidance on how best to integrate family-centered care into survivorship programs effectively. This aspect has also been underscored in other studies focusing on chronic illnesses such as cardiovascular disease [39] or dementia [40], emphasizing the importance of caring for families as a unified unit.

This gap underscores the urgent need for further research and the development of evidence-based interventions and practices that recognize and address the interconnected needs of survivors and their families. By adopting a family-centered approach to cancer survivorship care, healthcare providers can better support survivors and their families in navigating the challenges of life after the end of treatments. This family-centered approach involves recognizing the family as an interactive unit or system, with assessment and intervention centered around aspects such as family structure, relationships, and functioning [16]. Moreover, efforts to enhance posttreatment care and information should prioritize strategies that promote open communication, shared decision-making, and mutual support within the family unit. This review enhances our comprehension of the posttreatment support requirements for survivors and family caregivers, emphasizing the crucial need for comprehensive, accessible, and continual support services that addresses the diverse physical, emotional, and practical dimensions of survivorship. Challenges persist beyond the completion of treatment, and influence various aspects of families’ lives, including physical health, psychological well-being, independence, sexuality, and social connectivity. These findings align with those of other studies [41, 42], which demonstrated that caregivers’ demographic characteristics and early caregiving experiences have a lasting impact on quality of life during the long-term phase of cancer. This finding underscores the enduring effects of caregiving and the necessity for health interventions to support family members not only throughout the caregiving journey but also beyond it.

In the context of rural care, recognizing the importance of digital health interventions for adult survivors and their families is crucial for understanding the holistic approach to survivorship care. These interventions could play a vital role in supporting survivors and their families through the complex journey of survivorship, addressing not only the late physical effects of cancer but also the accompanying emotional, psychological, and social challenges. A systematic review and meta-analysis aimed at evaluating the impact of digital health interventions on psychosocial outcomes in adult cancer patients and their families throughout the cancer trajectory confirmed that these interventions were effective at improving quality of life and reducing anxiety and depression symptoms in patients and families [43]. These promising findings show that by utilizing digital platforms, future interventions could offer accessible and convenient avenues for survivors and their families to access support, education, and resources tailored to their specific needs. Furthermore, integrating digital health psychosocial interventions into survivorship care could enhance continuity of care beyond the clinical setting, empowering patients and their families to actively participate in managing their well-being and navigating life after cancer treatment.

After undergoing cancer treatment, survivors and their partners often face challenges related to intimacy and sexuality [44, 45]. The late effects of treatment can significantly impact survivors’ feelings of intimacy and their ability to engage in sexual activity. Changes in body image, fatigue, pain, and hormonal fluctuations may contribute to decreased libido or sexual function, while psychological factors such as anxiety, depression, and fear of recurrence can also affect survivors’ sexual well-being [46, 47]. Despite these challenges, many survivors seek to reclaim intimacy and sexuality, explore ways to reconnect with their partners, and rediscover pleasure and intimacy in their relationships. Open communication, support from healthcare professionals, and access to resources for sexual health and relationship counselling are essential for helping survivors and their partners navigate these sensitive issues and achieve a fulfilling posttreatment sexual and intimate life.

In developing survivorship care plans, it is crucial to consider the needs of family members, who often shoulder significant burdens alongside cancer survivors [48, 49]. Practical challenges, such as navigating the complexities of the healthcare system, accessing essential support services, and managing the financial strains associated with treatment, can compound the already stressful experience for families. While survivors strive to establish a “new normal” following cancer treatment, it is important to recognize that caregivers may continue to grapple with emotional challenges long after their loved one completes treatment. Therefore, survivorship care plans must extend beyond individual survivors to encompass the holistic needs of the entire family unit. By addressing the practical difficulties and emotional strains faced by family members, healthcare providers can ensure that survivorship care plans are comprehensive and inclusive, offering tailored support and resources to help both survivors and their families navigate the complexities of life after cancer [24].

Finally, the present review sheds light on several key gaps concerning cancer survivors and their families’ posttreatment completion. Despite advances in cancer care, the willingness of cancer survivors and families to engage in self-management strategies is poorly understood, highlighting the need for further large-scale trials tailored to address the unique challenges faced by this population. Future research directions should prioritize investigating the relationship between late effects and quality of life in survivors and families, developing contextually and culturally appropriate interventions for rural cancer survivors, and evaluating the effectiveness of interventions targeting behavioral and psychosocial determinants of health outcomes. Furthermore, investigating the efficacy and quality of communication, education, and support among healthcare professionals could offer valuable perspectives on meeting the unaddressed needs of cancer survivors and their families during the posttreatment phase.

### Strength and limitations

The current review was conducted with primary studies while overlooking unpublished studies and gray literature. This may limit the comprehensiveness of the review and lead to the overlooking of valuable insights that could contribute to a more nuanced understanding of the research on posttreatment health interventions for adult survivors of cancer and their families. However, the search included four languages to provide an international view of the current research. Additionally, a range of key words were used to identify the survivorship phase to ensure that most of the current research was included. Furthermore, the geographic distribution of the included studies was limited to France, Australia, Canada, the UK, and the USA. This limited representation of countries suggests a potential bias in the available evidence, as experiences and healthcare systems may vary significantly across different regions and cultures. Consequently, the generalizability of the findings to diverse populations and healthcare contexts may be compromised.

## Conclusion

This integrative review provides insights into the complex landscape of posttreatment support requirements for cancer survivors and their family caregivers. This finding underscores the critical necessity for additional intervention research involving comprehensive, accessible, and supportive services that address the multifaceted dimensions of survivorship for the patient and family as a unit. Beyond the completion of treatment, families encounter a myriad of challenges impacting various aspects of their lives, including physical health, psychological well-being, sexuality, and the social dimension. By adopting a family-centered approach to survivorship care and prioritizing strategies promoting open communication and mutual support within the family unit, healthcare providers may support survivors and their families in navigating the challenges of survivorship beyond treatment completion.

## Data Availability

No datasets were generated or analysed during the current study.
